# MEDLINE citation tool accuracy: an analysis in two platforms

**DOI:** 10.5195/jmla.2024.1718

**Published:** 2024-04-01

**Authors:** Laurel Scheinfeld, Sunny Chung

**Affiliations:** 1 laurel.scheinfeld@stonybrook.edu, Senior Assistant Librarian, Health Sciences Library, Stony Brook University, Stony Brook, NY; 2 sunny.chung@stonybrook.edu, Senior Assistant Librarian, Health Sciences Library, Stony Brook University, Stony Brook, NY

**Keywords:** Auto-citation generator, librarians, information literacy, citation on demand, biomedical databases, PubMed, Ovid MEDLINE

## Abstract

**Background:**

Libraries provide access to databases with auto-cite features embedded into the services; however, the accuracy of these auto-cite buttons is not very high in humanities and social sciences databases.

**Case Presentation:**

This case compares two biomedical databases, Ovid MEDLINE and PubMed, to see if either is reliable enough to confidently recommend to students for use when writing papers. A total of 60 citations were assessed, 30 citations from each citation generator, based on the top 30 articles in PubMed from 2010 to 2020.

**Conclusions:**

Error rates were higher in Ovid MEDLINE than PubMed but neither database platform provided error-free references. The auto-cite tools were not reliable. Zero of the 60 citations examined were 100% correct. Librarians should continue to advise students not to rely solely upon citation generators in these biomedical databases.

## BACKGROUND

Librarians in academic health sciences libraries support research at their institutions in myriad ways [1]. The integrity of the research process is an essential element of research and librarians often include research integrity as part of their instruction [2]. Proper attribution of prior work is one component of trustworthy research and it is important for students to learn both the purpose and the mechanics of accurate citation [3]. Most academic libraries provide support for properly citing sources in the form of style manuals and online style guides, while some libraries provide additional services including tutorials, workshops and individual consultations [4]. Though some librarians may feel it is the responsibility of the course instructor to teach the mechanics of proper citation, others embrace a team approach among instructors and academic support centers on campus, including libraries and writing centers. Even without agreement on how much responsibility should fall to librarians to teach proper citing, students and faculty naturally look to us for help. Regardless of personal preferences, librarians will inevitably be approached for assistance with citations [5, 6].

Formatting citations is often considered frustrating as well as time consuming, and one of the last tasks completed in preparing a research project [7]. Software tools have been developed to alleviate some of the burden of applying the rules for proper citation in a particular style. These tools include free-standing online citation generators, citation generators within databases and discovery tools, and bibliographic management software that creates formatted references as one of its functions. Studies show that many students make use of these tools. [7,8,9,10].

The features and functions of these tools vary, but all are meant to generate accurately formatted citations. Accuracy is crucial for academic and research endeavors, but the reliability of citation generator tools has come into question. Several studies have shed light on the limitations and shortcomings of various citation resources, raising concerns among librarians. In a recent study of the accuracy of three free online citation generators (ZoteroBib, CiteMaker and Cite This For Me), Ho [11] found that all the citations generated had errors. Six sources taken from student papers were used to generate citations in each tool and the eighteen citations were analyzed for ten different errors. Based on the results, Ho concluded that none of the tools could produce perfect references. Users were advised to be prepared to correct common formatting errors such as capitalization, punctuation, and indentation [11]. In a separate investigation by Laing and James, the focus shifted to the accuracy of citations generated by EBSCO and Summon Discovery Services. The researchers examined sixty sources, including print books, eBooks, and journal articles. They generated citations in three different citation styles using both platforms. Out of the total 180 citations, EBSCO produced 86 correct citations, while Summon managed only 31. The authors cautioned that neither platform could reliably generate accurate citations[12]. In 2005 and 2012, Van Ullen and Kessler conducted studies examining the citation support available in various humanities and social science databases. Their analysis encompassed citations on demand and other citation assistance provided within the databases. Although a minor improvement was observed in the 2012 study compared to the earlier one, the number of errors was still considered unacceptably high. The researchers expressed their disappointment, stating that citation help in databases continued to be compiled with little attention to detail. [13,14]. Even bibliographic management software, with its robust features for importing and saving data and creating citations in dozens of styles had disappointing results when studied for accurate formatting. Kratochvil [15] compared the accuracy of citations generated by EndNote, Mendeley, RefWorks and Zotero. The four different software platforms produced citations with 882, 679, 937 and 575 errors, respectively, out of 1084 references. Multiple errors in each citation were deemed completely unacceptable, as citations are fundamentally reliant on accurate details [15]. Collectively, these studies paint a disconcerting picture of the current state of citation generators. Despite their convenience and widespread usage, these tools often fall short in producing accurate and reliable citations.

At Stony Brook University Health Sciences Library, we provide multiple publication and style manuals as part of our reference collection, and we regularly point students to the Library's online citation guide. Of the various citation styles, the American Psychological Association (APA), is by far the style that we are most frequently asked for help with. Among our various patrons, those from the School of Nursing, the School of Social Welfare, and the undergraduate Health Sciences major request the most help with APA style. They are often concerned about receiving lower marks on assignments for inaccurate citation formatting. We offer workshops on APA Style each semester at the Health Sciences Library. Registration and attendance have been high compared to other library workshops offered so it seems to fill a learning need. The workshops include instruction about the importance of accurate citation and provide guidance on the proper formatting of citations. The workshops lead to requests for additional assistance since they establish the library as a place to go for help with citing. Questions about the accuracy of tools that automatically generate citations frequently come up in workshops and consults. We have always encouraged patrons to learn the rules of the citation style they are writing in, and we caution them to carefully review citations created with any citation generator, based on the evidence in the literature showing poor accuracy for these tools.

In reviewing the previous literature, we found no assessment of citation tools in biomedical databases. Users may expect citation tools embedded in biomedical databases to be superior to citation tools in other databases. These platforms have sophisticated technology for creating highly complex searches [16] so users may assume they have a higher degree of accuracy in citations as well. It is important for librarians to know whether the same caution against relying on automatic citation tools also applies to the tools in more advanced medical databases. Our study examines the accuracy of the ‘citation on demand' tool in the PubMed and Ovid MEDLINE platforms. We specifically concentrated on APA Style and journal articles. This decision was made as these are the predominant style and source type requirements for assignments in the School of Nursing, School of Social Work, and the Health Sciences major at our institution. MEDLINE indexes a large number of psychology, psychiatry, social work and nursing journals. PubMed is freely available and frequently used by all health sciences students at our institution to search MEDLINE. Although Ovid MEDLINE may be utilized less frequently by students, it offers automatically generated citations in APA 7th edition style making it suitable to compare the same citations in both platforms.

## CASE PRESENTATION

### Study Preparation

To gather a convenience sample of journal article citations, PubMed's trending articles webpage <https://pubmed.ncbi.nlm.nih.gov/trending/> was utilized to gather articles for this analysis. It was suggested as a viable source of citations for bibliographic analysis by several colleagues on MedLibEd, an online forum for medical librarians. We chose to sort the articles by the ‘Best match' option to obtain a more varied sample than sorting by other available options (‘Most recent', ‘Publication date', ‘First author' or ‘Journal'). We then limited to articles published from 2010-2020 and collected the first 30 articles. There was one retracted article which we did not include. Authors then checked that all selected articles were available in Ovid MEDLINE as well.

Citations were generated in APA 7th edition style from both databases using the ‘citation on demand' or ‘auto-cite tool' and were then copied and pasted into a shared document. (see supplemental materials). The 7th edition of the APA Manual was published in late 2019 and data for this study was collected in 2022, which we felt provided ample time for databases to make updates.

### Data Collection

The sixty journal article citations were carefully reviewed by two independent reviewers. Discrepancies were resolved through discussion. Fourteen elements of each citation were checked for accuracy. The 14 elements analyzed for errors were chosen by reviewing a sample of APA style journal citations and listing all elements that reviewers determined could contain errors. Both format and content were analyzed as both are necessary for an accurate citation. The number of elements we reviewed varies from previous studies because our assessment was specific to journal articles in APA Style only. Previous research analyzed either a variety of source types [7] or a variety of citation styles [11], and more general categories were often used. For example, ‘Name of Source' is an element assessed in other citation accuracy studies whereas for this research there are three separate elements related to the name of the source (‘journal name not abbreviated', ‘journal name in title case, with correct punctuation', and ‘journal name italicized'). We felt this allowed for a more specific and comprehensive assessment, though it limits the ability to compare results directly with other studies.

**Figure 1:**
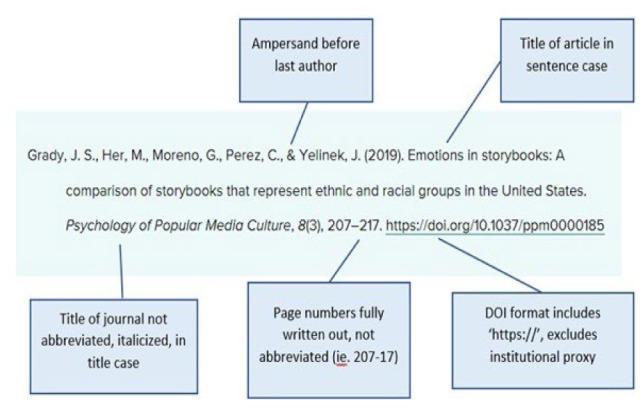
APA Style, 7th Edition Sample Journal Article Citation (Journal Article References, n.d.)

Reviewers relied on guidance from the APA Publication Manual and contacted the style experts at the American Psychological Association as needed for clarification on the written guide. See Figure 1 for a sample APA 7th edition journal article citation from the APA Manual.

The 14 elements checked for accuracy include:

- presence of a hanging indent
- correct author last name(s) followed by correct first and middle (if available) initials, with correct punctuation
- ampersand sign before the last author
- correct year of publication, with correct punctuation
- article title in sentence case, with correct punctuation
- journal name not abbreviated
- journal name in title case, with correct punctuation
- journal name italicized
- volume number correct, with correct punctuation
- volume number italicized
- issue number correct, with correct punctuation
- page numbers correct and correctly formatted
- DOI is included if available
- DOI formatted correctly.

**Figure 2:**
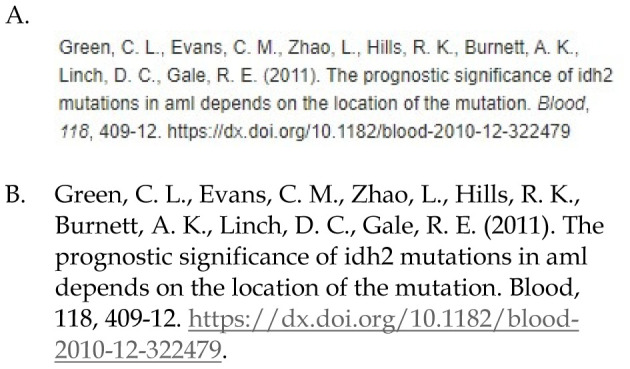
Comparison of sample journal article citation from Ovid MEDLINE citation generator (A) screenshot versus (B) same citation copied and pasted into a document using the ‘copy' button. Note journal name and volume number are not italicized in the copied and pasted citation.

Reviewers discovered that in Ovid MEDLINE there were differences between citations that were copied by highlighting the citation versus clicking the “copy” button. Using the copy button feature resulted in journal names and volume numbers not being italicized.(See Figure 2) Ultimately, reviewers assessed citations based on the copy button results since it is available and featured by the database. If a user were to manually type what they saw on screen into their document, or highlight the citation to copy it, the italicization would be correct, but we felt users were unlikely to do that when a simple click of the “copy” button is available.

Though effort was made to be thorough in choosing elements to review, two additional elements were not included in this assessment: font and line spacing. The reviewers found that when citations were copied and pasted, line spacing and fonts differed depending on the word processor used (Google Docs vs. Microsoft Word vs. Microsoft WordPad). It was beyond the scope of this study to assess accuracy for multiple document types so font and line spacing were excluded from the assessment.

Each reviewer assessed the 60 citations independently and entered findings into a screening spreadsheet which included a row for each citation and a column for each element of the citation. Elements found to be incorrect were designated with a ‘1'; correct elements were designated ‘0.' The sum of each row was used to calculate the total number of errors per citation, which could range from 0 to 14. (See supplemental materials). Results were compared and discrepancies were resolved through discussion and consensus.

## RESULTS

**Figure 3:**
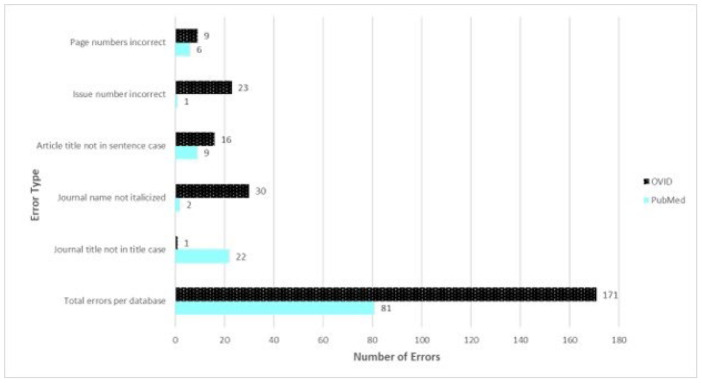
Elements with the highest number of errors and Total errors per database

**Figure 4:**
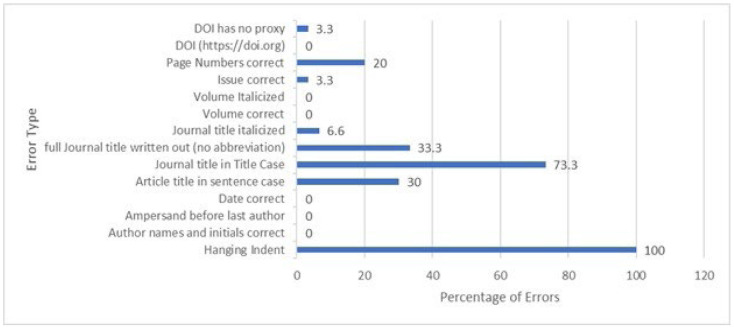
Percentage of errors in PubMed

**Figure 5:**
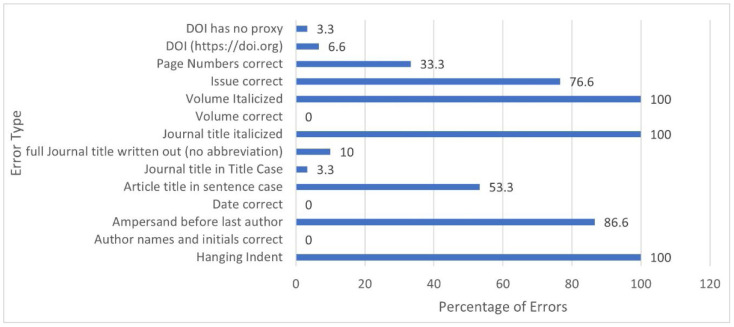
Percentage of errors in OVID

The total number of errors for both databases and the elements with the highest number of errors in each database are displayed in Figure 3. PubMed produced 81 total errors in the 30 citations, while Ovid MEDLINE produced 171. Errors in individual Ovid citations ranged from 4-9 with an average of 5.7 errors per citation, while the number of PubMed errors in individual citations ranged from 1-5; average 2.7 per citation. As mistakes per citation increase in the PubMed citations, there is a proportional increase in mistakes in the Ovid citations. This can be seen in Figures 4 and 5. No noticeable trends were noted in mistakes related to Journal, Publisher or Year.

The types of errors varied greatly by database. One of the frequent errors seen in PubMed was the journal name in the incorrect case. In 22 out of 30 PubMed citations, the journal name was in sentence case rather than the correct ‘title case.' Additionally, there were 3 PubMed citations in which the journal name consisted of only one word (i.e., Blood). In these cases, those elements were marked ‘correct,' though it is very possible that they would have been incorrect if the journal name consisted of multiple words. At variance with this finding were the Ovid MEDLINE citations, in which only one citation out of 30 was not generated in ‘title case.' When assessing correct italicizing of journal names, PubMed yielded errors in 2 citations while Ovid generated errors in all 30 citations. For the article title element, ‘sentence case' is the correct format; PubMed yielded 9 errors while Ovid yielded 16 errors. Ovid generated significantly more errors than PubMed in displaying issue numbers. Ovid consistently left out issue numbers from the auto-generated citations, totaling 23 errors. The Ovid citations that were correct were those from journals that do not use issue numbers. When displaying page numbers, PubMed produced 6 errors, while Ovid produced 9. Six of the errors in both databases involved online-only journals that use article numbers instead of page numbers. When that is the case, the APA guidelines direct authors to insert the word “Article” in front of the article's number, instead of the standard page numbers. These errors in the citations were due to the absence of the word “Article.” The remaining 3 errors in the Ovid database were due to the abbreviation of page numbers (i.e., displaying 561-8, rather than 561-568). A major source of errors common to both databases was the lack of a hanging indent. All 60 citations were missing this element. OVID also consistently omitted the required ampersand sign (&) before the last author's name, though both platforms otherwise had authors names and initials correct 100% of the time. We looked at punctuation within each element (author names, date, article title, journal, DOI). Neither database had punctuation errors and they both also did a good job of generating accurate publication dates and volume numbers. In our analysis, the DOI (digital object identifier), was generated accurately in both platforms, but subsequently we noticed that if using PubMed through our institution's website, the proxy link would be included within the DOI, which would be inaccurate according to APA guidelines. When patrons access PubMed through an institutional proxy, they need to check that the DOI does not contain the proxy link.

## DISCUSSION

Each citation tool has its strengths, but neither is accurate enough to recommend using as a sole source of APA Style 7th edition citations. PubMed produced citations with an average of 2.7 errors. The common errors in PubMed citations were lack of the hanging indent, incorrect case for journal names and article titles, and using abbreviations in journal names. Ovid MEDLINE citations produced an average of 5.7 errors. Common Ovid errors were the lack of hanging indent, lack of ampersand in author element when there is more than one author, incorrect case for article titles, lack of italicization for journal names and volume numbers, lack of issue numbers, and incorrect page number format. Ovid's auto-cite tool generated more than double the number of errors as PubMed amongst all 14 of the criteria examined. A positive finding is that punctuation is reliable in the citation tools of these biomedical databases. That was not the case for other automatically generated citations in tools previously studied [7, 11] Despite this, we cannot say that databases with more sophisticated searching capability contain better citation tools based on our results. Though punctuation and content were accurate most of the time, the amount of formatting errors are problematic enough that these tools cannot be relied upon solely. They require the user to have knowledge of proper APA Style to make necessary corrections. Therefore, we recommend that students learn how to cite references correctly and that they regularly consult style manuals. If they choose to utilize a citation generator for convenience, the resulting citations should be checked and edited for accuracy, specifically focusing on the formatting errors noted above in each platform.

There was no noticeable correlation between the number of mistakes and Journal or Publisher, but the sample size is insufficient to definitively conclude the absence of any association. Though we often hear that database citation tools can only be as accurate as the data imported from the publisher, that does not explain why the same reference displays with different formatting errors depending on the database. For example, in most of the 30 PubMed citations we looked at, the journal name was correctly formatted in Title Case, whereas in the same 30 citations generated in Ovid MEDLINE, most of the journal titles were not formatted in Title Case. Conversely, in Ovid MEDLINE, most Journal titles and issue numbers were correctly formatted in italics, whereas in PubMed most were not. This shows that the capability exists for the tools to manipulate data into the correct capitalization and italicization, but the capability appears to be underutilized and arbitrary. Whether this is due to lack of awareness or limited resources on the part of database creators is worth discussing with vendors. In addition, in Ovid MEDLINE, the discrepancy between the generated citations seen on the screen and those same citations copied and pasted into a document seem simple to correct and would decrease the error rate substantially.

We reached out to the NLM and Ovid to share our concerns. Both were very responsive and eager to improve the citation tools. Amanda Sawyer of NLM shared that “there are certain rules that–in our experience–machines cannot produce with 100% accuracy. For example, there is no way to systematically capitalize proper nouns, acronyms, chemical formulas, abbreviations, etc., that is completely reliable and also complies with all rules.” Both NLM and Ovid asked for specific examples and expressed their intentions to investigate further and make improvements if possible. Librarians who would like to see these tools improved are encouraged to contact their database vendor representatives as well.

The conclusions of this report are consistent with previous research that the accuracy of auto citation generators is currently unacceptably low. Some limitations are the small sample size and testing a single citation style and resource type. Results cannot be directly compared to previous studies since methods of analysis have varied. Future research in this area could involve steps toward creating a standardized method of assessing the accuracy of reference entries.

We noticed a significant number of articles in our sample with electronic article numbers, rather than page numbers. Given the steady move away from print journals and towards increased electronic publishing of journals, this can be expected to increase. The citation generators in these two databases do not appear programmed to handle the format of the electronic article numbers correctly (see sample reference entry below from our analysis). In the sample reference entry, the word “Article” before the article number has not been inserted. Librarians are advised to familiarize themselves with the correct formatting of these electronic article numbers and to share this information when teaching APA citation style.

Salvo, F., Moore, N., Arnaud, M., Robinson, P., Raschi, E., De Ponti, F., Begaud, B., Pariente, A. (2016). Addition of dipeptidyl peptidase-4 inhibitors to sulphonylureas and risk of hypoglycaemia: systematic review and meta-analysis. BMJ, 353, i2231. https://dx.doi.org/10.1136/bmj.i2231.

The accuracy of word font and line spacing were difficult to assess for this analysis. We found that the font and spacing changed depending on whether we pasted the citations into Microsoft Word, Microsoft WordPad, or Google Docs. Additionally, when pasting a citation from the Ovid MEDLINE database into Google Docs, a different font and line spacing resulted depending on whether the user copied the citation by highlighting it, or by using the available “copy” button. See supplementary material for examples. In the end, it was decided not to include font and spacing in our assessment of accuracy. There are specific recommendations for fonts and spacing in the APA manual and it's important for librarians to warn patrons to carefully check their formatting of these elements and to include the guidelines for correct fonts and spacing in any educational workshops on APA Style.

Though many software tools continue to be available to assist with creating a reference list, auto-cite tools in these two biomedical databases did not produce accurate citations in APA format. If students wish to use them for convenience, librarians can caution them to check for and correct common formatting errors. There is less need to check for content or punctuation errors as those elements were generally accurate. Being familiar with the correct formatting will enable students to more easily correct automatically generated citations. Based on this case study, Librarians still have a crucial role to play in teaching both the importance and the specifics of proper citation as part of research integrity and information literacy.

## DATA AVAILABILITY STATEMENT

All data from this investigation are contained in the appendices. There are no additional data associated with this article.

## AUTHOR CONTRIBUTIONS

Laurel Scheinfeld: conceptualization, methodology, formal analysis, investigation, writing-original draft, writing-review & editing, visualization, project administration; Sunny Chung: investigation, formal analysis, writing-original draft, writing-review & editing, visualization

## SUPPLEMENTAL FILES

**Appendix A:** Reviewer 1 Citation Screening Sheet

**Appendix B:** Reviewer 2 Citation Screening Sheet

**Appendix C:** PubMed Citations

**Appendix D:** Ovid MEDLINE Citations

**Appendix E:** Descriptive Breakdown of Articles

**Appendix F:** Google Doc Fonts

**Appendix G:** Word Fonts

**Appendix H:** WordPad Fonts
