# Supplementary material for: MEDLINE citation tool accuracy: an analysis in two platforms
Source: J Med Libr Assoc. 2024 May 22;112(2):133–9. doi: 10.5195/jmla.2024.1718 (PMC11305470; doi:10.5195/jmla.2024.1718)

**______________________________________________________________________________**

- **PubMed 9/9/22 Trending Articles, sorted by ‘best match’, first 30 articles that are in both databases, 2010-2020, no retracted articles**

# PubMed Citations - Each citation is Copy/Pasted followed by screenshot(s)

#

Eich, C., Bleckmann, A., & Schwarz, S. K. (2007). Percussion pacing--an almost forgotten procedure for haemodynamically unstable bradycardias? A report of three case studies and review of the literature. *British journal of anaesthesia*, *98*(4), 429–433. <https://doi-org.proxy.library.stonybrook.edu/10.1093/bja/aem007>


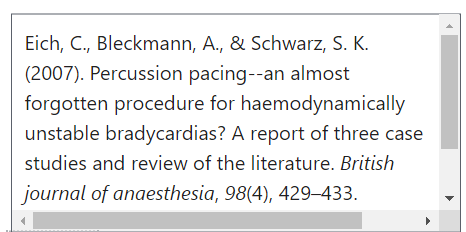


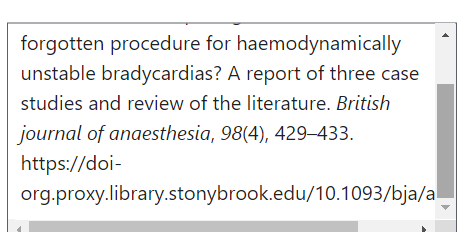


White, R. C., & Remington, A. (2019). Object personification in autism: This paper will be very sad if you don't read it. *Autism : the international journal of research and practice*, *23*(4), 1042–1045. <https://doi.org/10.1177/1362361318793408>


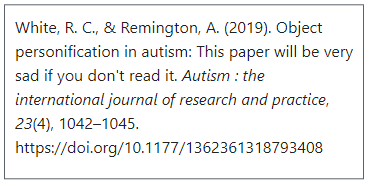


Green, C. L., Evans, C. M., Zhao, L., Hills, R. K., Burnett, A. K., Linch, D. C., & Gale, R. E. (2011). The prognostic significance of IDH2 mutations in AML depends on the location of the mutation. *Blood*, *118*(2), 409–412. <https://doi.org/10.1182/blood-2010-12-322479>


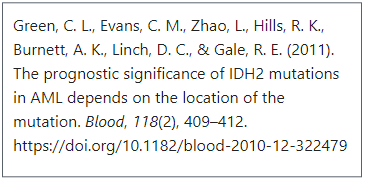


Rafiq, S., Zia, S., Ijaz, M. J., Shahid, H. A., & Adeel, M. (2018). Role of Weight-Bearing Exercises in the Treatment of Post-Menopausal Osteoporosis. *Journal of the College of Physicians and Surgeons--Pakistan : JCPSP*, *28*(2), 122–125. https://doi-org.proxy.library.stonybrook.edu/10.29271/jcpsp.2018.02.122


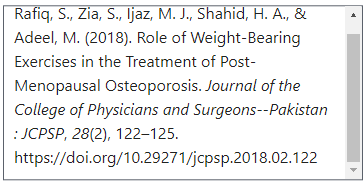


Hu, Y., Li, J., Li, J., Zhang, F., Wang, J., Mo, M., & Liu, Y. (2019). Biocontrol efficacy of Pseudoxanthomonas japonensis against Meloidogyne incognita and its nematostatic metabolites. *FEMS microbiology letters*, *366*(2), 10.1093/femsle/fny287. <https://doi.org/10.1093/femsle/fny287>


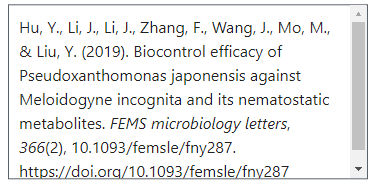


Jansen van Rensburg, M. J., Whitelaw, A. C., & Elisha, B. G. (2012). Genetic basis of rifampicin resistance in methicillin-resistant Staphylococcus aureus suggests clonal expansion in hospitals in Cape Town, South Africa. *BMC microbiology*, *12*, 46. <https://doi.org/10.1186/1471-2180-12-46>

**
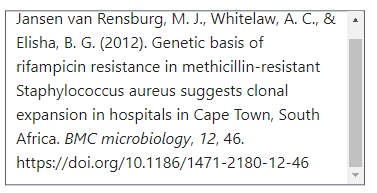
**

Chen, H., Zhou, W., Ruan, Y., Yang, L., Xu, N., Chen, R., Yang, R., Sun, J., & Zhang, Z. (2018). Reversal of angiotensin ll-induced β-cell dedifferentiation via inhibition of NF-κb signaling. *Molecular medicine (Cambridge, Mass.)*, *24*(1), 43. <https://doi.org/10.1186/s10020-018-0044-3>


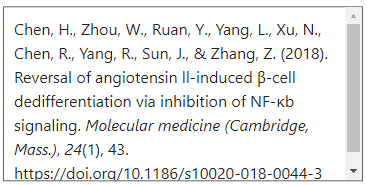


Angelelli, P., Marinelli, C. V., De Salvatore, M., & Burani, C. (2017). Morpheme-based Reading and Spelling in Italian Children with Developmental Dyslexia and Dysorthography. *Dyslexia (Chichester, England)*, *23*(4), 387–405. <https://doi.org/10.1002/dys.1554>


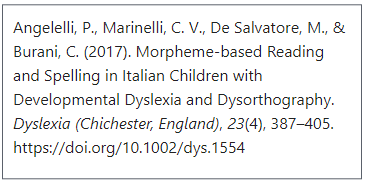


Sales, R. R., Belisário, A. R., Faria, G., Mendes, F., Luizon, M. R., & Viana, M. B. (2020). Functional polymorphisms of BCL11A and HBS1L-MYB genes affect both fetal hemoglobin level and clinical outcomes in a cohort of children with sickle cell anemia. *Annals of hematology*, *99*(7), 1453–1463. <https://doi.org/10.1007/s00277-020-04079-2>


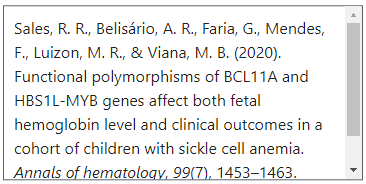
 (Screenshot could not fit the whole citation)
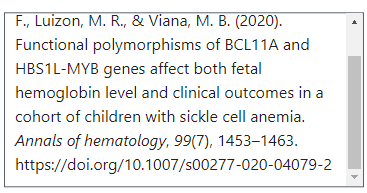


Mikityansky, I., Zager, E. L., Yousem, D. M., & Loevner, L. A. (2012). MR Imaging of the brachial plexus. *Magnetic resonance imaging clinics of North America*, *20*(4), 791–826. <https://doi.org/10.1016/j.mric.2012.08.003>


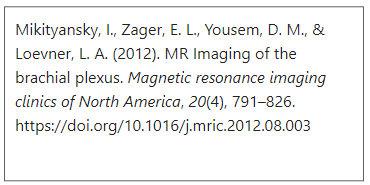


Benn, B. S., Parikh, M., Tsau, P. H., Seeley, E., & Krishna, G. (2019). Using a Dedicated Interventional Pulmonology Practice Decreases Wait Time Before Treatment Initiation for New Lung Cancer Diagnoses. *Lung*, *197*(2), 249–255. <https://doi.org/10.1007/s00408-019-00207-6>


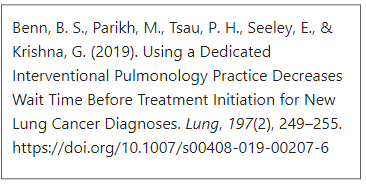


Boopathy, S., Silvas, T. V., Tischbein, M., Jansen, S., Shandilya, S. M., Zitzewitz, J. A., Landers, J. E., Goode, B. L., Schiffer, C. A., & Bosco, D. A. (2015). Structural basis for mutation-induced destabilization of profilin 1 in ALS. *Proceedings of the National Academy of Sciences of the United States of America*, *112*(26), 7984–7989. <https://doi.org/10.1073/pnas.1424108112>


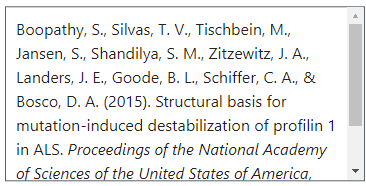
 (Screenshot could not fit the whole citation)


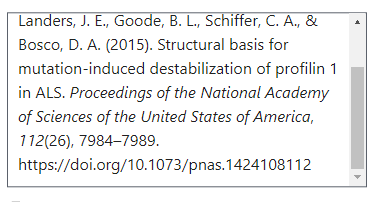


Nuzzo J. L. (2020). The Case for Retiring Flexibility as a Major Component of Physical Fitness. *Sports medicine (Auckland, N.Z.)*, *50*(5), 853–870. <https://doi.org/10.1007/s40279-019-01248-w>


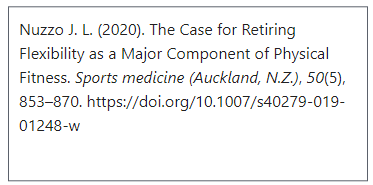


Bohnert, K., Dorizas, A., Lorenc, P., & Sadick, N. S. (2019). Randomized, Controlled, Multicentered, Double-Blind Investigation of Injectable Poly-L-Lactic Acid for Improving Skin Quality. *Dermatologic surgery : official publication for American Society for Dermatologic Surgery [et al.]*, *45*(5), 718–724. <https://doi.org/10.1097/DSS.0000000000001772>


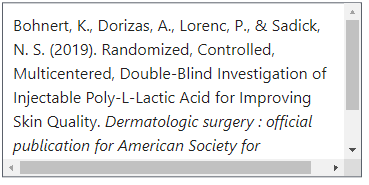
 (Screenshot could not fit the whole citation)


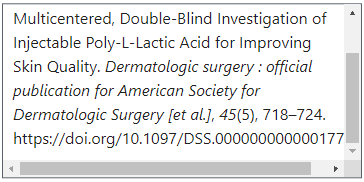


de la Torre, X., Colamonici, C., Iannone, M., Jardines, D., Molaioni, F., & Botrè, F. (2020). Detection of clostebol in sports: Accidental doping?. *Drug testing and analysis*, *12*(11-12), 1561–1569. <https://doi.org/10.1002/dta.2951>


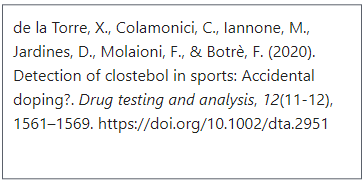


Srisawat, N., & Kellum, J. A. (2020). The Role of Biomarkers in Acute Kidney Injury. *Critical care clinics*, *36*(1), 125–140. <https://doi.org/10.1016/j.ccc.2019.08.010>


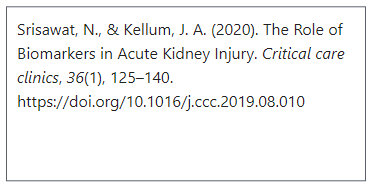


Salvo, F., Moore, N., Arnaud, M., Robinson, P., Raschi, E., De Ponti, F., Bégaud, B., & Pariente, A. (2016). Addition of dipeptidyl peptidase-4 inhibitors to sulphonylureas and risk of hypoglycaemia: systematic review and meta-analysis. *BMJ (Clinical research ed.)*, *353*, i2231. <https://doi.org/10.1136/bmj.i2231>


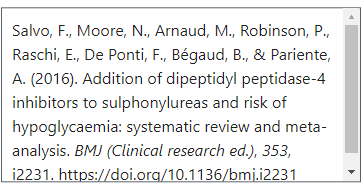
(Screenshot could not fit the whole citation)


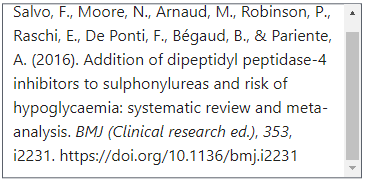


Stovitz, S. D., Banack, H. R., & Kaufman, J. S. (2018). Paediatric obesity appears to lower the risk of diabetes if selection bias is ignored. *Journal of epidemiology and community health*, *72*(4), 302–308. <https://doi.org/10.1136/jech-2017-209985>


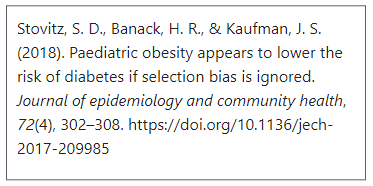


Sinatra, S. T., Oschman, J. L., Chevalier, G., & Sinatra, D. (2017). Electric Nutrition: The Surprising Health and Healing Benefits of Biological Grounding (Earthing). *Alternative therapies in health and medicine*, *23*(5), 8–16.


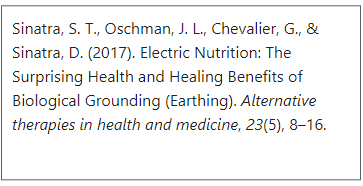


Marican, A., & Durán-Lara, E. F. (2018). A review on pesticide removal through different processes. *Environmental science and pollution research international*, *25*(3), 2051–2064. https://doi.org/10.1007/s11356-017-0796-2


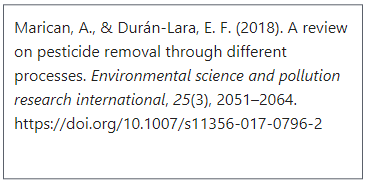


Sinclair R. D. (2018). Female pattern hair loss: a pilot study investigating combination therapy with low-dose oral minoxidil and spironolactone. *International journal of dermatology*, *57*(1), 104–109. <https://doi.org/10.1111/ijd.13838>


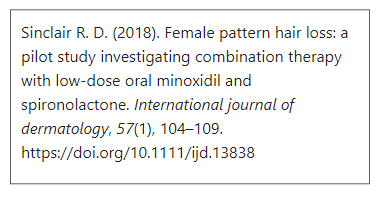


Lima Júnior, E. M., Moraes Filho, M. O., Forte, A. J., Costa, B. A., Fechine, F. V., Alves, A., Moraes, M., Rocha, M., Silva Júnior, F. R., Soares, M., Bezerra, A. N., Martins, C. B., & Mathor, M. B. (2020). Pediatric Burn Treatment Using Tilapia Skin as a Xenograft for Superficial Partial-Thickness Wounds: A Pilot Study. *Journal of burn care & research : official publication of the American Burn Association*, *41*(2), 241–247. <https://doi.org/10.1093/jbcr/irz149>


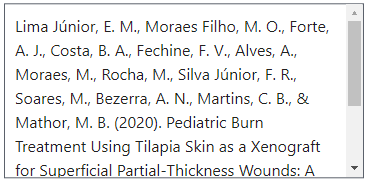
(Screenshot could not fit the whole citation)


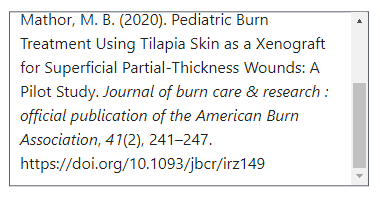


McRae, G., Payne, A., Zelt, J. G., Scribbans, T. D., Jung, M. E., Little, J. P., & Gurd, B. J. (2012). Extremely low volume, whole-body aerobic-resistance training improves aerobic fitness and muscular endurance in females. *Applied physiology, nutrition, and metabolism = Physiologie appliquee, nutrition et metabolisme*, *37*(6), 1124–1131. <https://doi.org/10.1139/h2012-093>


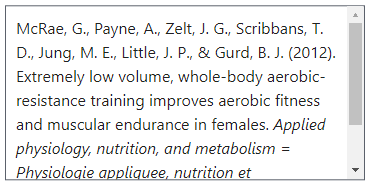
(Screenshot could not fit the whole citation)


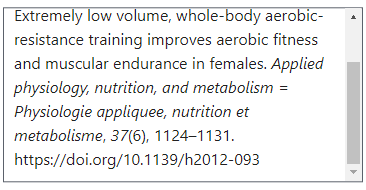


Blanchflower D. G. (2021). Is happiness U-shaped everywhere? Age and subjective well-being in 145 countries. *Journal of population economics*, *34*(2), 575–624. <https://doi.org/10.1007/s00148-020-00797-z>


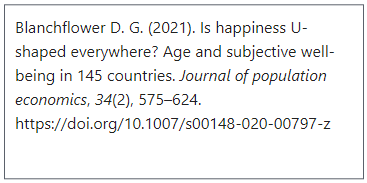


Ernst E. (2010). Deaths after chiropractic: a review of published cases. *International journal of clinical practice*, *64*(8), 1162–1165. <https://doi.org/10.1111/j.1742-1241.2010.02352.x>


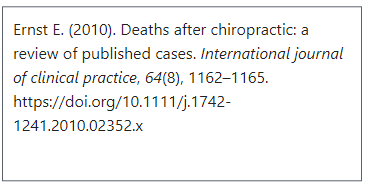


Hill, S. J., Rolland, T., Adelmant, G., Xia, X., Owen, M. S., Dricot, A., Zack, T. I., Sahni, N., Jacob, Y., Hao, T., McKinney, K. M., Clark, A. P., Reyon, D., Tsai, S. Q., Joung, J. K., Beroukhim, R., Marto, J. A., Vidal, M., Gaudet, S., Hill, D. E., … Livingston, D. M. (2014). Systematic screening reveals a role for BRCA1 in the response to transcription-associated DNA damage. *Genes & development*, *28*(17), 1957–1975. <https://doi.org/10.1101/gad.241620.114>


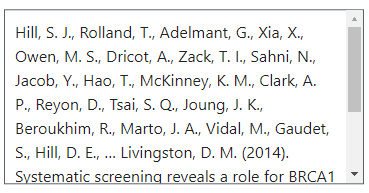
(Screenshot could not fit the whole citation)


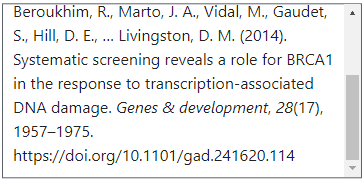


MacLean, E., Kohli, M., Weber, S. F., Suresh, A., Schumacher, S. G., Denkinger, C. M., & Pai, M. (2020). Advances in Molecular Diagnosis of Tuberculosis. *Journal of clinical microbiology*, *58*(10), e01582-19. <https://doi.org/10.1128/JCM.01582-19>


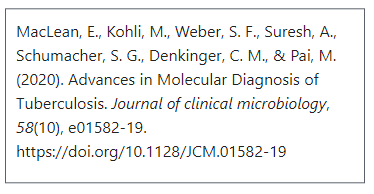


Huang, Y., Cai, X., Mai, W., Li, M., & Hu, Y. (2016). Association between prediabetes and risk of cardiovascular disease and all cause mortality: systematic review and meta-analysis. *BMJ (Clinical research ed.)*, *355*, i5953. <https://doi.org/10.1136/bmj.i5953>


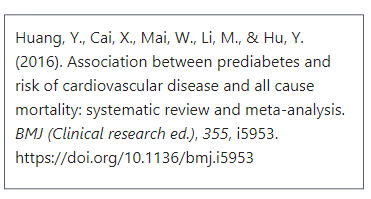


Bourassa, K. J., Caspi, A., Harrington, H., Houts, R., Poulton, R., Ramrakha, S., & Moffitt, T. E. (2020). Intimate partner violence and lower relationship quality are associated with faster biological aging. *Psychology and aging*, *35*(8), 1127–1139. <https://doi.org/10.1037/pag0000581>


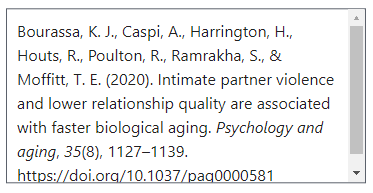


Garcia-Alvarez, M., Marik, P., & Bellomo, R. (2014). Stress hyperlactataemia: present understanding and controversy. *The lancet. Diabetes & endocrinology*, *2*(4), 339–347. <https://doi.org/10.1016/S2213-8587(13)70154-2>


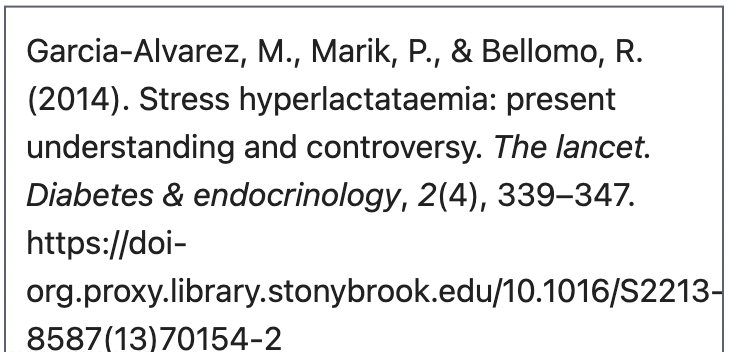

Supplement: Supplementary file 3 — Appendix C: PubMed Citations [file jmla-112-2-133-s03.docx]
