# Supplementary material for: MEDLINE citation tool accuracy: an analysis in two platforms
Source: J Med Libr Assoc. 2024 May 22;112(2):133–9. doi: 10.5195/jmla.2024.1718 (PMC11305470; doi:10.5195/jmla.2024.1718)

# OVID Medline 30 Citations 9/9/22 - Each citation is Copied/Pasted followed by screenshot

Ovid using “copy” button

Eich, C., Bleckmann, A., Schwarz, S. K. (2007). Percussion pacing--an almost forgotten procedure for haemodynamically unstable bradycardias? a report of three case studies and review of the literature. British Journal of Anaesthesia, 98, 429-33.

Ovid manually copy

Eich, C., Bleckmann, A., Schwarz, S. K. (2007). Percussion pacing--an almost forgotten procedure for haemodynamically unstable bradycardias? a report of three case studies and review of the literature. *British Journal of Anaesthesia*, *98*, 429-33.

White, R. C., Remington, A. (2019). Object personification in autism: this paper will be very sad if you don't read it. Autism, 23, 1042-1045. <https://dx.doi.org/10.1177/1362361318793408>


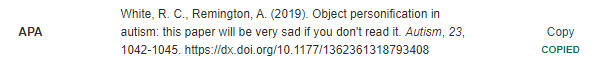


Green, C. L., Evans, C. M., Zhao, L., Hills, R. K., Burnett, A. K., Linch, D. C., Gale, R. E. (2011). The prognostic significance of idh2 mutations in aml depends on the location of the mutation. Blood, 118, 409-12. <https://dx.doi.org/10.1182/blood-2010-12-322479>


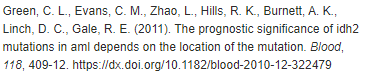


Rafiq, S., Zia, S., Ijaz, M. J., Shahid, H. A., Adeel, M. (2018). Role of weight-bearing exercises in the treatment of post-menopausal osteoporosis. Jcpsp, Journal of the College of Physicians & Surgeons - Pakistan, 28, 122-125. <https://dx.doi.org/10.29271/jcpsp.2018.02.122>


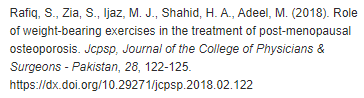


Hu, Y., Li, J., Li, J., Zhang, F., Wang, J., Mo, M., Liu, Y. (2019). Biocontrol efficacy of pseudoxanthomonas japonensis against meloidogyne incognita and its nematostatic metabolites. FEMS Microbiology Letters, 366, <https://dx.doi.org/10.1093/femsle/fny287>


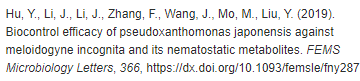


Jansen van Rensburg, M. J., Whitelaw, A. C., Elisha, B. G. (2012). Genetic basis of rifampicin resistance in methicillin-resistant staphylococcus aureus suggests clonal expansion in hospitals in cape town, south africa. BMC Microbiology, 12, 46. <https://dx.doi.org/10.1186/1471-2180-12-46>


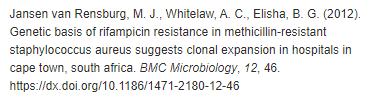


Chen, H., Zhou, W., Ruan, Y., Yang, L., Xu, N., Chen, R., Yang, R., Sun, J., Zhang, Z. (2018). Reversal of angiotensin ll-induced beta-cell dedifferentiation via inhibition of nf-κb signaling. Molecular Medicine, 24, 43. <https://dx.doi.org/10.1186/s10020-018-0044-3>


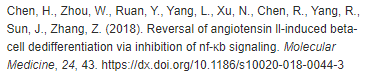


Angelelli, P., Marinelli, C. V., De Salvatore, M., Burani, C. (2017). Morpheme-based reading and spelling in italian children with developmental dyslexia and dysorthography. Dyslexia: the Journal of the British Dyslexia Association, 23, 387-405. <https://dx.doi.org/10.1002/dys.1554>


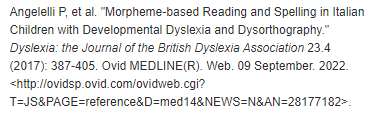


Sales, R. R., Belisario, A. R., Faria, G., Mendes, F., Luizon, M. R., Viana, M. B. (2020). Functional polymorphisms of bcl11a and hbs1l-myb genes affect both fetal hemoglobin level and clinical outcomes in a cohort of children with sickle cell anemia. Annals of Hematology, 99, 1453-1463. <https://dx.doi.org/10.1007/s00277-020-04079-2>


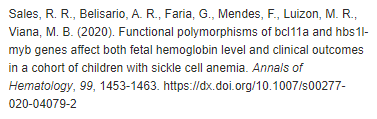


Mikityansky, I., Zager, E. L., Yousem, D. M., Loevner, L. A. (2012). Mr imaging of the brachial plexus. Magnetic Resonance Imaging Clinics of North America, 20, 791-826. <https://dx.doi.org/10.1016/j.mric.2012.08.003>


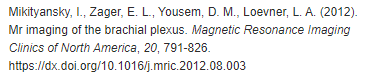


Benn, B. S., Parikh, M., Tsau, P. H., Seeley, E., Krishna, G. (2019). Using a dedicated interventional pulmonology practice decreases wait time before treatment initiation for new lung cancer diagnoses. Lung, 197, 249-255. <https://dx.doi.org/10.1007/s00408-019-00207-6>


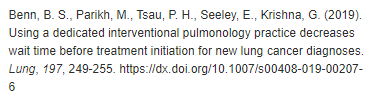


Boopathy, S., Silvas, T. V., Tischbein, M., Jansen, S., Shandilya, S. M., Zitzewitz, J. A., Landers, J. E., Goode, B. L., Schiffer, C. A., Bosco, D. A. (2015). Structural basis for mutation-induced destabilization of profilin 1 in als. Proceedings of the National Academy of Sciences of the United States of America, 112, 7984-9. <https://dx.doi.org/10.1073/pnas.1424108112>


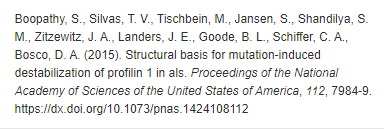


Nuzzo, J. L. (2020). The case for retiring flexibility as a major component of physical fitness. Sports Medicine, 50, 853-870. https://dx.doi.org/10.1007/s40279-019-01248-w


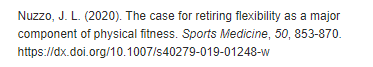


Bohnert, K., Dorizas, A., Lorenc, P., Sadick, N. S. (2019). Randomized, controlled, multicentered, double-blind investigation of injectable poly-l-lactic acid for improving skin quality. Dermatologic Surgery, 45, 718-724. <https://dx.doi.org/10.1097/DSS.0000000000001772>


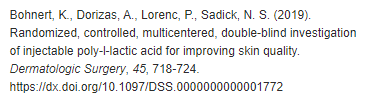


de la Torre, X., Colamonici, C., Iannone, M., Jardines, D., Molaioni, F., Botre, F. (2020). Detection of clostebol in sports: accidental doping? Drug Testing & Analysis, 12, 1561-1569. <https://dx.doi.org/10.1002/dta.2951>.


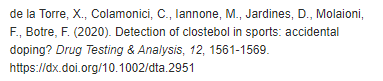


Srisawat, N., Kellum, J. A. (2020). The role of biomarkers in acute kidney injury. Critical Care Clinics, 36, 125-140. https://dx.doi.org/10.1016/j.ccc.2019.08.010


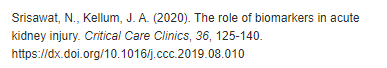


Salvo, F., Moore, N., Arnaud, M., Robinson, P., Raschi, E., De Ponti, F., Begaud, B., Pariente, A. (2016). Addition of dipeptidyl peptidase-4 inhibitors to sulphonylureas and risk of hypoglycaemia: systematic review and meta-analysis. BMJ, 353, i2231. <https://dx.doi.org/10.1136/bmj.i2231>


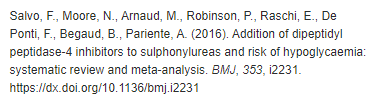


Stovitz, S. D., Banack, H. R., Kaufman, J. S. (2018). Paediatric obesity appears to lower the risk of diabetes if selection bias is ignored. Journal of Epidemiology & Community Health, 72, 302-308. <https://dx.doi.org/10.1136/jech-2017-209985>


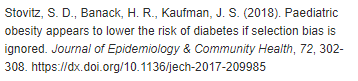


Sinatra, S. T., Oschman, J. L., Chevalier, G., Sinatra, D. (2017). Electric nutrition: the surprising health and healing benefits of biological grounding (earthing). Alternative Therapies in Health & Medicine, 23(5), 8-16. Retrieved from <http://ovidsp.ovid.com/ovidweb.cgi?T=JS&PAGE=reference&D=med14&NEWS=N&AN=28987038>.


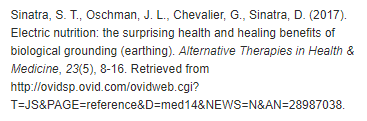


Marican, A., Duran-Lara, E. F. (2018). A review on pesticide removal through different processes. Environmental Science & Pollution Research, 25, 2051-2064. <https://dx.doi.org/10.1007/s11356-017-0796-2>


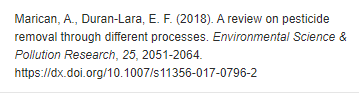


Sinclair, R. D. (2018). Female pattern hair loss: a pilot study investigating combination therapy with low-dose oral minoxidil and spironolactone. International Journal of Dermatology, 57, 104-109. <https://dx.doi.org/10.1111/ijd.13838>


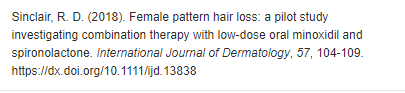


Lima Junior, E. M., Moraes Filho, M. O., Forte, A. J., Costa, B. A., Fechine, F. V., Alves, A. P. N. N., Moraes, M. E. A., Rocha, M. B. S., Silva Junior, F. R., Soares, Bezerra, A. N., Martins, C. B., Mathor, M. B. (2020). Pediatric burn treatment using tilapia skin as a xenograft for superficial partial-thickness wounds: a pilot study. Journal of Burn Care & Research, 41, 241-247. <https://dx.doi.org/10.1093/jbcr/irz149>


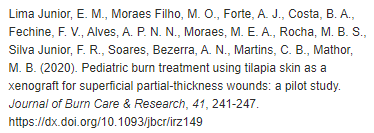


McRae, G., Payne, A., Zelt, J. G., Scribbans, T. D., Jung, M. E., Little, J. P., Gurd, B. J. (2012). Extremely low volume, whole-body aerobic-resistance training improves aerobic fitness and muscular endurance in females. Applied Physiology, Nutrition, & Metabolism = Physiologie Appliquee, Nutrition et Metabolisme, 37, 1124-31. <https://dx.doi.org/10.1139/h2012-093>


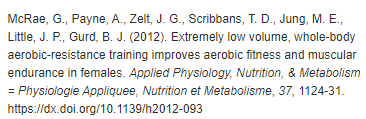


Blanchflower, D. G. (2021). Is happiness u-shaped everywhere? age and subjective well-being in 145 countries. Journal of Population Economics, 34, 575-624. <https://dx.doi.org/10.1007/s00148-020-00797-z>


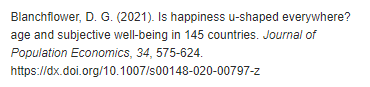


Ernst, E. (2010). Deaths after chiropractic: a review of published cases. International Journal of Clinical Practice, 64, 1162-5. <https://dx.doi.org/10.1111/j.1742-1241.2010.02352.x>


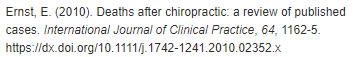


Hill, S. J., Rolland, T., Adelmant, G., Xia, X., Owen, M. S., Dricot, A., Zack, T. I., Sahni, N., Jacob, Y., Hao, T., McKinney, K. M., Clark, A. P., Reyon, D., Tsai, S. Q., Joung, J. K., Beroukhim, R., Marto, J. A., Vidal, M., Gaudet, S., Hill, D. E., Livingston, D. M. (2014). Systematic screening reveals a role for brca1 in the response to transcription-associated dna damage. Genes & Development, 28, 1957-75. <https://dx.doi.org/10.1101/gad.241620.114>


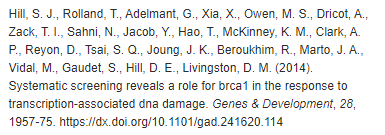


MacLean, E., Kohli, M., Weber, S. F., Suresh, A., Schumacher, S. G., Denkinger, C. M., Pai, M. (2020). Advances in molecular diagnosis of tuberculosis. Journal of Clinical Microbiology, 58, <https://dx.doi.org/10.1128/JCM.01582-19>


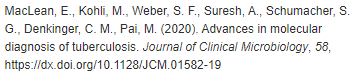


Huang, Y., Cai, X., Mai, W., Li, M., Hu, Y. (2016). Association between prediabetes and risk of cardiovascular disease and all cause mortality: systematic review and meta-analysis. BMJ, 355, i5953. <https://dx.doi.org/10.1136/bmj.i5953>


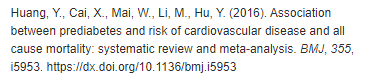


Bourassa, K. J., Caspi, A., Harrington, H., Houts, R., Poulton, R., Ramrakha, S., Moffitt, T. E. (2020). Intimate partner violence and lower relationship quality are associated with faster biological aging. Psychology & Aging, 35, 1127-1139. <https://dx.doi.org/10.1037/pag0000581>


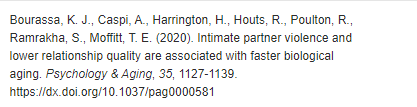


Garcia-Alvarez, M., Marik, P., Bellomo, R. (2014). Stress hyperlactataemia: present understanding and controversy. The Lancet Diabetes & Endocrinology, 2, 339-347. <https://dx.doi.org/10.1016/S2213-8587(13)70154-2>

.


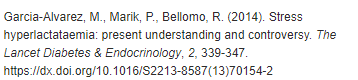

Supplement: Supplementary file 4 — Appendix D: Ovid MEDLINE Citations [file jmla-112-2-133-s04.docx]
