# Supplementary material for: MEDLINE citation tool accuracy: an analysis in two platforms
Source: J Med Libr Assoc. 2024 May 22;112(2):133–9. doi: 10.5195/jmla.2024.1718 (PMC11305470; doi:10.5195/jmla.2024.1718)
Supplement: Supplementary file 6 — Appendix F: Google Doc Fonts [file jmla-112-2-133-s06.docx]

**PubMed Copy button - Roboto 12, Line Spacing 1.15**

White, R. C., & Remington, A. (2019). Object personification in autism: This paper will be very sad if you don't read it. *Autism : the international journal of research and practice*, *23*(4), 1042–1045. <https://doi-org.proxy.library.stonybrook.edu/10.1177/1362361318793408>

**PubMed Copy/Paste - Roboto 12, 1.15**

White, R. C., & Remington, A. (2019). Object personification in autism: This paper will be very sad if you don't read it. *Autism : the international journal of research and practice*, *23*(4), 1042–1045. <https://doi-org.proxy.library.stonybrook.edu/10.1177/1362361318793408>

**OvidMedline Copy button - Roboto 12, 1.15**

White, R. C., Remington, A. (2019). Object personification in autism: this paper will be very sad if you don't read it. Autism, 23, 1042-1045. https://dx.doi.org/10.1177/1362361318793408

**OvidMedline Copy/Paste - Arial 9. 1.5**

| White, R. C., Remington, A. (2019). Object personification in autism: this paper will be very sad if you don't read it. *Autism*, *23*, 1042-1045. https://dx.doi.org/10.1177/1362361318793408 |
| --- |
