# Supplementary material for: MEDLINE citation tool accuracy: an analysis in two platforms
Source: J Med Libr Assoc. 2024 May 22;112(2):133–9. doi: 10.5195/jmla.2024.1718 (PMC11305470; doi:10.5195/jmla.2024.1718)
Supplement: Supplementary file 7 — Appendix G: Word Fonts [file jmla-112-2-133-s07.docx]

**PubMed Copy Button - Segoe UI 11, Line spacing 1.08**

White, R. C., & Remington, A. (2019). Object personification in autism: This paper will be very sad if you don't read it. *Autism : the international journal of research and practice*, *23*(4), 1042–1045. <https://doi-org.proxy.library.stonybrook.edu/10.1177/1362361318793408>

**PubMed Copy/Paste –**  **Segoe UI 11, Line spacing 1.08**

White, R. C., & Remington, A. (2019). Object personification in autism: This paper will be very sad if you don't read it. *Autism : the international journal of research and practice*, *23*(4), 1042–1045. <https://doi-org.proxy.library.stonybrook.edu/10.1177/1362361318793408>

**Ovid Medline Copy/Paste – Helvetica 9, 1.08**
White, R. C., Remington, A. (2019). Object personification in autism: this paper will be very sad if you don't read it. *Autism*, *23*, 1042-1045. <https://dx.doi.org/10.1177/1362361318793408>

**Ovid Medline Copy Button** – **Helvetica 9, 1.08**
White, R. C., Remington, A. (2019). Object personification in autism: this paper will be very sad if you don't read it. Autism, 23, 1042-1045. https://dx.doi.org/10.1177/1362361318793408
